# The Frequency of Exacerbations in Patients with COPD and Their Nutritional Status: A Multicenter Study †

**DOI:** 10.3390/medicina62061121

**Published:** 2026-06-09

**Authors:** Ceren Degirmenci, Maide Gozde Inam, Ozge Oral Tapan, Aytekin Idikut, Silam Yesilyurt, Fatih Tekin, Serife Nur Ozturk, Muge Gencer Tuluy, Ugur Fidan, Seyma Tunc, Nazli Cetin, Neslihan Kose Kabil, Zeynep Yilmaz Kaya

**Affiliations:** 1 Department of Chest Diseases, Mugla Training and Research Hospital, 48000 Mugla, Türkiye; 2 Vagelos College of Physicians and Surgeons, Columbia University Irving Medical Center, New York, NY 10032, USA; mgozdebagci@hotmail.com; 3 Department of Chest Diseases, Faculty of Medicine, Training and Research Hospital, Mugla Sitki Kocman University, 48000 Mugla, Türkiye; ozgeoral@mu.edu.tr; 4 Department of Chest Diseases, Bartin State Hospital, 74100 Bartın, Türkiye; aytekinidikut@gmail.com; 5 Department of Chest Diseases, Sincan Training and Research Hospital, 06949 Ankara, Türkiye; silam32@hotmail.com; 6 Department of Chest Diseases, Van Training and Research Hospital, 65300 Van, Türkiye; ffatihtekin@hotmail.com; 7 Department of Chest Diseases, Gaziantep City Hospital, 27470 Gaziantep, Türkiye; nur.palaz@gmail.com (S.N.O.); zeynep96yilmaz@gmail.com (Z.Y.K.); 8 Department of Chest Diseases, Izmir Buca Seyfi Demirsoy Training and Research Hospital, 35390 İzmir, Türkiye; dr.muge.gencer@gmail.com; 9 Department of Chest Diseases, Batman Kozluk State Hospital, 72400 Batman, Türkiye; dr-ugurfidan@hotmail.com; 10 Department of Chest Diseases, Kastamonu Training and Research Hospital, 37150 Kastamonu, Türkiye; seyma1100@gmail.com; 11 Department of Chest Diseases, Afyonkarahisar State Hospital, 03030 Afyonkarahisar, Türkiye; nazlicetin@yandex.com; 12 Department of Chest Diseases, Faculty of Medicine, Yalova University, 77200 Yalova, Türkiye; neslihankose.nks@gmail.com

**Keywords:** COPD, exacerbation, geriatric nutritional risk index, nutrition, prognostic nutritional index

## Abstract

*Background and Objectives*: Nutritional impairment and systemic inflammation contribute to disease progression and poor outcomes in Chronic Obstructive Pulmonary Disease (COPD). The geriatric-nutritional-risk-index (GNRI) and prognostic-nutritional-index (PNI) are practical markers reflecting both nutritional and immune status. In elderly COPD patients, malnutrition-related exacerbations often worsen quality of life and increase hospitalization. Identifying reliable predictors of exacerbation risk is therefore important for improving disease management. This study evaluated the association between GNRI, PNI and exacerbation frequency across different age groups in COPD. *Materials and Methods*: This multicenter retrospective study included 302 patients with COPD from 10 medical centers. All patients were classified as GOLD Group-E according to exacerbation history. Demographic characteristics, pulmonary function tests, Charlson-Comorbidity-Index (CCI), pharmacological treatments, dyspnea scores, and annual exacerbation frequency were obtained from hospital databases. Laboratory parameters including complete blood count, C-reactive protein, albumin, and total protein were recorded. GNRI, PNI, and neutrophil-to-lymphocyte ratio (NLR) were calculated to evaluate nutritional and inflammatory status. *Results*: The mean age of participants was 67.9 ± 9.6 years and 26.5% were female. Elderly patients had significantly higher CCI scores, longer disease duration, greater cumulative smoking exposure, and more frequent exacerbations than younger patients (*p* < 0.001). Pulmonary function parameters were significantly lower in the elderly group, while long-term oxygen therapy and nebulizer use were more common (*p* < 0.001). Baseline and exacerbation NLR levels were higher in elderly patients, whereas GNRI and PNI values were lower during both stable disease and exacerbation periods. Patients with more than four exacerbations per year had significantly higher NLR and lower GNRI values. *Conclusions*: Elderly COPD patients in GOLD Group-E demonstrate marked inflammatory and nutritional burden. Lower PNI values were independently associated with increased annual exacerbation frequency, while lower GNRI values were observed in patients with greater inflammatory and nutritional burden. Routine immune-nutritional assessment may improve risk stratification and help identify patients who could benefit from early multidisciplinary management.

## 1. Introduction

Chronic Obstructive Pulmonary Disease (COPD) is a leading cause of global mortality and represents a major public health burden, particularly in low- and middle-income countries [1,2]. Acute exacerbations, defined as sudden worsening of respiratory symptoms requiring additional treatment, are pivotal events that accelerate lung function decline, impair quality of life, and increase hospitalization and healthcare costs [3,4]. Therefore, identifying reliable predictors of exacerbation risk remains a central objective in COPD management [5].

Malnutrition is a common and often underrecognized comorbidity in COPD, driven by increased metabolic demand, reduced dietary intake, systemic inflammation, and catabolic processes [6,7,8]. Impaired nutritional status contributes to muscle wasting, immune dysfunction, reduced exercise capacity, and worse clinical outcomes, particularly in aging populations [9]. In the elderly, this nutritional decline is often exacerbated by age-related sarcopenia and immunosenescence, making this demographic particularly vulnerable to the adverse effects of respiratory impairment. Accordingly, simple and objective tools for assessing nutritional risk may provide clinically meaningful prognostic information.

The geriatric nutritional risk index (GNRI) and the prognostic nutritional index (PNI) are practical, cost-effective markers reflecting both nutritional and immune-inflammatory status. GNRI, calculated using anthropometric parameters and serum albumin levels, has been associated with increased mortality and adverse outcomes in elderly populations [10,11,12,13]. PNI, derived from serum albumin concentration and lymphocyte count, has demonstrated prognostic value in surgical and oncological settings and has recently been suggested as a potential indicator of exacerbation risk in elderly COPD patients [14,15,16,17,18]. However, evidence evaluating these indices across different age groups in COPD remains limited.

Since nutritional indices like GNRI and PNI are inherently linked to systemic inflammation through serum albumin levels, a comprehensive assessment must also account for broader inflammatory markers. Systemic inflammation plays a central role in COPD pathogenesis and progression. The Systemic Immune-Inflammation Index (SII), calculated from neutrophil, lymphocyte, and platelet counts, reflects the balance between inflammatory activation and immune response [7]. Although elevated SII levels have been associated with disease severity, their independent contribution to exacerbation burden in clinically stable COPD has not been fully clarified.

However, it is not yet established whether nutritional indices independently predict exacerbation burden after adjustment for lung function and symptom severity, or whether this association is modified by age. We hypothesized that impaired nutritional status, assessed by GNRI and PNI, is independently associated with increased annual exacerbation frequency in patients with COPD, even after controlling for established clinical predictors.

In light of the recent GOLD 2026 recommendations, which emphasize the clinical impact of even a single moderate exacerbation by defining it as Group E, identifying biomarkers that predict exacerbation risk is increasingly vital. Accordingly, the primary objective of this study was to determine whether GNRI and PNI independently predict annual exacerbation frequency across age-stratified COPD populations—all classified as GOLD Group E—using multivariable regression analysis. The secondary objective was to evaluate the interaction between systemic inflammation, assessed by SII, nutritional status, and exacerbation burden.

## 2. Materials and Methods

### 2.1. Study Population

This multicenter retrospective observational study was conducted to evaluate the relationship between nutritional and inflammatory biomarkers and COPD exacerbation frequency. Following ethical approval, medical records of patients diagnosed with COPD who experienced exacerbations and visited pulmonary clinics at 10 participating centers between 1 January 2024 and 31 December 2024 were retrospectively retrieved from institutional databases. These data were extracted and recorded into a standardized electronic system by the participating physicians at each hospital, with each center contributing a minimum of 20 patients to ensure a balanced multicenter distribution. Ethical approval for the study was obtained from the Mugla Sitki Kocman University Ethics Committee (project number: 250050/80, date: 11 April 2025).

Inclusion criteria were defined according to the guidelines of the Global Initiative for Chronic Obstructive Lung Disease (GOLD) as patients diagnosed with COPD for at least one year and having at least one pulmonary clinic visit, emergency department visit, or hospitalization due to COPD exacerbation within the past year [3]. COPD exacerbation was defined as an acute worsening of symptoms diagnosed by a pulmonologist according to GOLD criteria. Stable COPD was defined as the absence of acute exacerbation requiring clinic visit, emergency visit, hospitalization, systemic corticosteroid, or antibiotic treatment within the preceding 4 weeks.

Demographic data, comorbidities, Charlson Comorbidity Index, pulmonary function test parameters, complete blood count parameters during both the stable COPD visit and the exacerbation period, as well as albumin, total protein, and CRP levels, were recorded separately. Furthermore, the COPD Assessment Test (CAT) and modified Medical Research Council (mMRC) dyspnea scores were assessed for all patients. Patients with missing data were excluded from the study.

**Figure 1:**
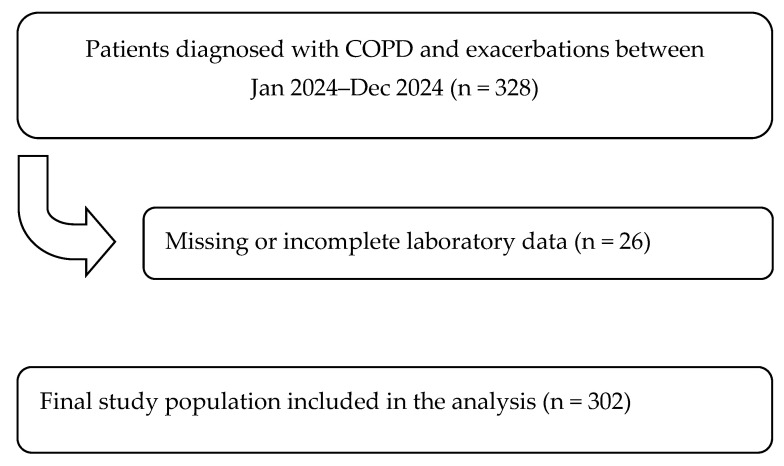
Flow chart of the study population selection and exclusion criteria.

**Figure 2:**
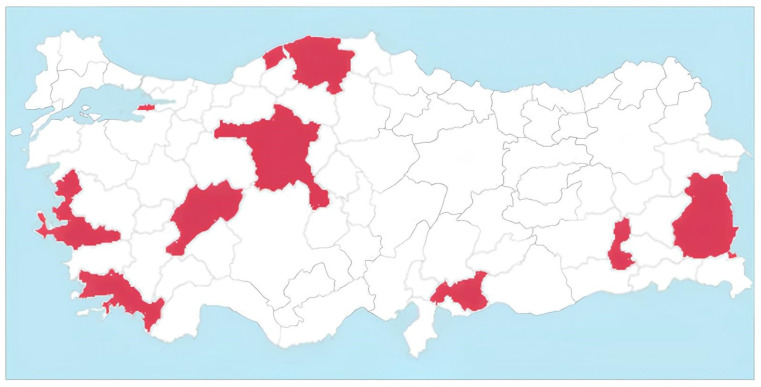
The representation of the centers included in the study. The areas marked in red indicate the provinces in Türkiye where patient data were included.

The sample size was calculated using G*Power 3.1.9.7 software based on an independent groups *t*-test design. The study was planned with an initial minimum sample size requirement derived from preliminary power considerations. However, the final analysis included 302 patients, which substantially exceeded the minimum required number (Figure 1 and Figure 2). This larger-than-planned sample size enhanced the statistical reliability, precision of estimates, and overall statistical power of the study.

### 2.2. Inflammation and Nutrition-Based Indicators

To evaluate systemic inflammation and nutritional status, a panel of blood-based biomarkers and indices was calculated. Hematological parameters, including total leukocyte, neutrophil, lymphocyte, and platelet counts, were obtained during both stable and exacerbation periods. Serum biochemical markers, including C-reactive protein (CRP), albumin, and total protein levels, were also recorded.

Systemic inflammation and nutritional status were assessed using validated indices derived from hematological and biochemical parameters. The SII was calculated by multiplying the platelet count by the neutrophil-to-lymphocyte ratio, as shown in Equation (1). Nutritional status was further evaluated using the PNI, defined in Equation (2), while the NLR was calculated from absolute neutrophil and lymphocyte counts (Equation (3)). Finally, GNRI was determined by incorporating serum albumin levels and anthropometric parameters, as presented in Equation (4).

SII = (Platelet count × neutrophil count)/Lymphocyte count(1)

PNI = (10 × Serum Albumin (g/dL)) + (0.005 × Total Lymphocyte Count (/mm^3^)) (2)

NLR = Neutrophil count/Lymphocyte count(3)

GNRI = (1.489 × Serum Albumin (g/L)) + (41.7 × Actual Body Weight/Ideal Body Weight)(4)

### 2.3. Statistical Analysis

All statistical analyses were conducted using SPSS version 25.0 (IBM Corp., Armonk, NY, USA). Continuous variables were expressed as mean ± standard deviation (SD), while categorical variables were summarized as frequencies and percentages. The distribution of data was assessed using the Shapiro–Wilk test. Comparisons between independent groups were performed using the Pearson chi-square test for categorical variables and the independent samples *t*-test or Mann–Whitney U test for continuous variables, as appropriate. Paired comparisons between stable and exacerbation periods were conducted using the paired *t*-test or Wilcoxon signed-rank test.

Inflammatory and nutritional indices were analyzed at both baseline and during exacerbations, with subgroup analyses performed according to age and exacerbation frequency. Statistical significance was defined as a *p*-value < 0.05.

A multivariable negative binomial regression model was employed to identify independent predictors of annual exacerbation frequency. Variables included in the multivariable model were selected based on their clinical relevance, previously established association with COPD exacerbation risk in the literature [3,18,19] and statistical significance in univariable analyses. Age, gender, FEV_1_, and CAT score were included in the model as clinically relevant covariates. To ensure robustness of the findings, model adequacy and specification were evaluated by testing for overdispersion and assessing goodness-of-fit statistics (χ^2^/df and pseudo-R^2^).

## 3. Results

A total of 302 patients were included in the study, with 103 participants younger than 65 years and 199 participants aged 65 years and older. The mean (±SD) age was 68 ± 10 years, and 26% of the participants were female. In accordance with the GOLD 2026 report, the entire study population was classified as Group E, as all participants had a history of at least one clinically significant exacerbation within the previous year. No significant differences were found in gender or BMI distribution between the age groups (*p* = 0.640 and *p* = 0.951, respectively). The demographic and clinical characteristics of all subjects are presented in Table 1.

Clinical parameters, including CAT score, pack-years, CCI, mMRC score, and duration of diagnosis, were significantly higher in the older age group (*p* < 0.001 for all). Additionally, annual exacerbation frequency was significantly higher in participants elder than 65 years (2.43 ± 1.77 vs. 3.46 ± 2.50, *p* < 0.001).

Pulmonary function test results indicated significantly lower values in the ≥65 age group for FEV_1_% predicted (48.27 ± 17.69 vs. 54.55 ± 18.40, *p* = 0.004), FEV_1_ (1.31 ± 0.56 L vs. 1.73 ± 0.70 L, *p* < 0.001), FVC% predicted (65.11 ± 19.82 vs. 70.88 ± 19.38, *p* = 0.017), FVC (2.29 ± 0.85 L vs. 2.82 ± 0.95 L, *p* < 0.001), and FEV_1_/FVC ratio (57.18 ± 10.21 vs. 60.4 ± 9.83, *p* = 0.003).

Regarding stable-period biomarkers, serum CRP (7.06 ± 14.83 vs. 4.76 ± 8.73, *p* = 0.019) was significantly higher, while serum albumin (38.49 ± 5.44 vs. 40.47 ± 5.10, *p* = 0.002) was significantly lower in the older group. No significant differences were observed in neutrophil count, lymphocyte count, platelet count, or total serum protein levels between the two age groups during the stable period (Table 1).

During exacerbation periods, lymphocyte count (*p* = 0.016), platelet count (*p* = 0.048), serum CRP (*p* = 0.023), and serum albumin (*p* = 0.001) differed significantly between age groups, with the older group consistently demonstrating more altered inflammatory marker profiles. Medication usage patterns are presented in Table 1. The use of long-term oxygen therapy (LTOT) and nebulizer treatment was significantly more prevalent in the ≥65 age group (*p* < 0.001 for both).

Inflammatory and nutritional indices revealed significant differences in baseline and exacerbation values of PNI (*p* = 0.002 and *p* < 0.001, respectively), NLR (*p* = 0.040 and *p* = 0.005, respectively), and GNRI (*p* = 0.017 and *p* = 0.028, respectively), with older patients demonstrating poorer nutritional status and higher inflammatory responses during both stable and exacerbation periods (Table 2).

In the overall patient cohort (n = 302), SII was significantly higher during exacerbation (2113.29 ± 2211.63) compared with baseline (1246.69 ± 1404.92, *p* < 0.001). Similarly, NLR was higher in the exacerbation period (7.89 ± 7.52) than at baseline (4.55 ± 4.12, *p* < 0.001). In contrast, both nutritional indices were significantly lower during exacerbation: PNI (38.31 ± 5.44 vs. 39.17 ± 5.40, *p* < 0.001) and GNRI (107.07 ± 13.33 vs. 108.36 ± 13.35, *p* < 0.001).

**Figure 3:**
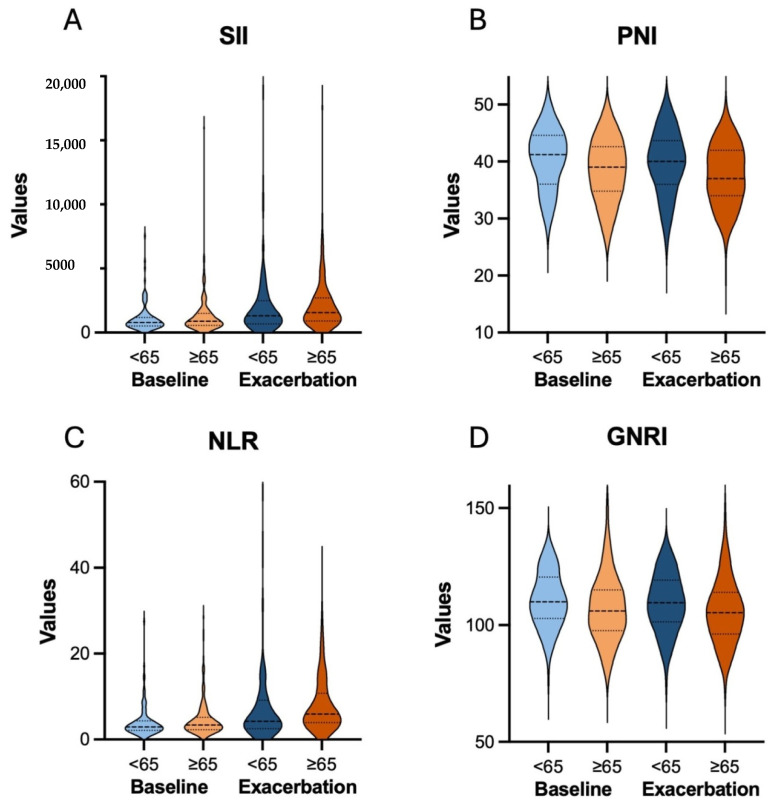
Distribution of inflammatory and nutritional indices according to age group (<65 and ≥65 years) and exacerbation status in patients with COPD. Violin plots show SII (**A**), PNI (**B**), NLR (**C**), and GNRI (**D**) values at baseline and during exacerbation. Inflammatory markers (SII and NLR) increased during exacerbation, whereas nutritional indices (PNI and GNRI) showed a decreasing trend. Dashed lines indicate the median and interquartile range.

Subgroup analysis by age demonstrated consistent findings, with SII and NLR values significantly increasing during exacerbation in both age groups (*p* < 0.001 for all). Additionally, both PNI and GNRI values significantly decreased during exacerbation, with a more pronounced decline in PNI observed in patients aged ≥65 years (*p* < 0.001) (Figure 3).

Patients with more than two exacerbations demonstrated significantly higher exacerbation-period SII and NLR values compared to those with two or fewer exacerbations (*p* = 0.010 and *p* < 0.001, respectively). Furthermore, baseline and exacerbation-period PNI levels were significantly lower in the >2 exacerbation group (*p* < 0.001 and *p* = 0.011, respectively), indicating poorer nutritional status. As shown in Table 3, no significant differences were observed in GNRI values between the groups.

In the entire study population, a multivariable negative binomial regression analysis was performed to identify independent predictors of annual exacerbation frequency (Table 4). The model demonstrated that lower PNI was significantly associated with an increased risk of exacerbation (estimate = −0.0141, 95% CI: −0.026 to −0.001, *p* = 0.034). Similarly, higher NLR was an independent predictor of exacerbation burden (estimate = 0.0271, 95% CI: 0.012 to 0.042, *p* < 0.001).

Decline in pulmonary function, as assessed by FEV_1_ (% predicted), was also strongly associated with higher exacerbation rates (estimate = −0.0136, 95% CI: −0.018 to −0.009, *p* < 0.001). Moreover, CAT score emerged as a significant predictor (estimate = 0.0312, 95% CI: 0.022 to 0.041, *p* < 0.001), indicating that greater symptom burden was linked to increased exacerbation risk. In the multivariable negative binomial regression analysis, lower baseline PNI and higher baseline NLR remained independently associated with increased annual exacerbation frequency after adjustment for age, gender, FEV_1_%, and CAT score.

In contrast, neither age (estimate = −0.0006, 95% CI: −0.015 to 0.014, *p* = 0.880) nor gender (estimate = −0.0131, 95% CI: −0.150 to 0.085, *p* = 0.865) showed significant associations with exacerbation frequency in the fully adjusted model. The model demonstrated good fit, with no evidence of overdispersion (χ^2^/df = 0.783), and acceptable explanatory capacity (pseudo R^2^ = 0.477).

## 4. Discussion

This multicenter study demonstrates that impaired nutritional status is independently associated with increased annual exacerbation frequency in patients with COPD, even after adjustment for lung function and symptom burden. Specifically, lower PNI and higher NLR values emerged as independent predictors of exacerbation burden in multivariable negative binomial regression analysis. In contrast, chronological age was not independently associated with exacerbation frequency after full adjustment, suggesting that biological determinants—such as systemic inflammation and immune-nutritional imbalance—may be more clinically relevant than age alone in exacerbation risk stratification.

Our findings extend previous observations linking malnutrition to adverse COPD outcomes by demonstrating that objective nutritional indices provide additional prognostic information beyond established predictors such as FEV_1_ and CAT score [14,18]. Although earlier studies have suggested that low PNI is associated with worse prognosis in elderly COPD populations, most were limited by single-center designs or lacked comprehensive adjustment for clinical confounders. By incorporating age stratification and multivariable modeling, our results indicate that the relationship between nutritional impairment and exacerbation burden is strong across different age groups. Although GNRI values were lower in elderly patients and during exacerbation periods, GNRI was not independently associated with annual exacerbation frequency after multivariable adjustment. Therefore, GNRI may primarily reflect the overall nutritional burden of COPD rather than serving as an independent prognostic marker for exacerbation frequency in this cohort.

According to the GOLD 2026 report, even a single moderate exacerbation is sufficient to classify a patient into Group E, emphasizing the clinical weight of any symptomatic instability. Our study cohort, consisting entirely of Group E patients, confirms that this group represents a high-risk population where immune-nutritional depletion is already prevalent. However, although the threshold for Group E has been simplified in recent guidelines, our findings demonstrate that patients with a higher exacerbation burden (e.g., >2 per year) exhibit even more profound alterations in PNI and NLR. This suggests that while all Group E patients are at risk, immune-nutritional indices can help identify a ‘very high-risk’ subgroup within the E category. However, our findings should not be interpreted as a proposal for a modification of the GOLD classification system. Rather, these results suggest that routinely available immune-nutritional markers may provide additional prognostic information within the already high-risk Group E population. Whether such subclassification has direct therapeutic or management implications remains uncertain and requires validation in prospective longitudinal studies.

Systemic inflammation is a central driver of COPD progression and exacerbation susceptibility. In our cohort, both SII and NLR increased significantly during exacerbation episodes across all age groups, reflecting an acute shift toward heightened inflammatory activation and relative lymphocyte suppression. Importantly, patients with frequent exacerbations demonstrated higher exacerbation-period SII and NLR values, suggesting that an exaggerated inflammatory response may contribute to recurrent clinical instability rather than representing a purely transient phenomenon.

Although SII levels were significantly elevated during exacerbation episodes and in patients with more frequent exacerbations, SII did not remain an independent predictor in the fully adjusted regression model. Therefore, SII should be interpreted primarily as a marker associated with systemic inflammatory burden rather than as an independent prognostic factor for exacerbation frequency. Residual confounding from unmeasured variables may also have contributed to the observed associations. These findings are consistent with previous reports linking elevated inflammatory markers to worse COPD outcomes while further clarifying their relative prognostic contributions in multivariable modeling [20,21,22].

The inverse relationship observed between nutritional indices and inflammatory markers supports the concept of a bidirectional interaction between malnutrition and systemic inflammation. Hypoalbuminemia and lymphocyte depletion—key components of PNI—may reflect chronic inflammatory activation and immune dysregulation. This interaction may generate a self-perpetuating cycle in which inflammation exacerbates nutritional deterioration, and nutritional impairment reduces immune resilience, thereby increasing susceptibility to recurrent exacerbations.

Interventional evidence further supports the biological plausibility of our findings. Previous studies have shown that nutritional supplementation in COPD patients may improve body composition, exercise capacity, and inflammatory profiles [23,24,25,26,27]. Randomized controlled trials conducted during acute exacerbations have demonstrated preservation of muscle strength with high-protein nutritional support [28], while meta-analyses in stable COPD populations have reported improvements in body weight, lean mass, and functional capacity following targeted supplementation [29,30]. Moreover, recent systematic reviews have suggested potential benefits of micronutrient and antioxidant supplementation on lung function parameters and exacerbation outcomes [31]. Although our study was not designed to evaluate therapeutic interventions, these data collectively indicate that nutritional optimization may represent a modifiable component of exacerbation risk. Given that all patients in our cohort belong to the high-risk GOLD Group E, our results suggest that targeted nutritional interventions could be a vital, non-pharmacological strategy to reduce the recurrent stability issues and systemic inflammation characteristic of this specific phenotype.

From a clinical perspective, routinely available laboratory-derived indices such as PNI and NLR may offer practical and cost-effective tools for identifying patients at higher risk of frequent exacerbations. Unlike complex scoring systems or advanced imaging modalities, these indices can be easily calculated from standard blood tests and may be particularly valuable in resource-limited settings. Incorporating nutritional evaluation into routine COPD management may facilitate earlier identification of vulnerable patients and support multidisciplinary interventions aimed at stabilizing disease trajectory.

Several limitations should be acknowledged. First, the retrospective design limits control over potential confounders and precludes causal inference. Although multivariable regression analysis was performed to adjust for key clinical variables, residual confounding from unmeasured factors—such as detailed body composition parameters, dietary intake, socioeconomic status, and medication adherence—cannot be entirely excluded. Accordingly, inflammatory markers such as SII should be interpreted cautiously, particularly when independent associations are not consistently maintained after multivariable adjustment. Second, exacerbation frequency was determined from documented clinical encounters, which may underestimate milder events not resulting in healthcare contact; however, this approach likely captures clinically significant exacerbations more reliably. In addition, the inclusion of only GOLD Group E patients may limit the generalizability of the findings to patients with milder COPD phenotypes or lower exacerbation risk. Third, although GNRI and PNI are validated and objective indices, they do not provide a comprehensive assessment of muscle mass, sarcopenia, or functional nutritional capacity. More detailed nutritional and body composition analyses were beyond the scope of this multicenter registry-based study. Finally, laboratory measurements obtained during exacerbation episodes were not collected at standardized time points. Hematological and inflammatory parameters were retrospectively obtained from available test results during the exacerbation period, regardless of the exact timing of blood sampling. Consequently, some blood samples may have been collected after the initiation of treatments such as corticosteroids or antibiotics, which may have influenced inflammatory and hematological parameters.

## 5. Conclusions

In conclusion, impaired nutritional status was closely associated with exacerbation burden in COPD. Lower PNI and higher NLR independently predicted annual exacerbation frequency beyond established determinants such as airflow limitation and symptom severity, whereas lower GNRI values appeared to reflect greater overall nutritional impairment and disease burden. These findings highlight the potential value of integrating routine nutritional assessment into COPD risk stratification and management strategies. Overall, our findings underscore that lower PNI and higher NLR are robust, independent predictors of annual exacerbation burden in COPD, regardless of airflow limitation or symptom severity. These results highlight the potential value of integrating routine immune-nutritional assessment into COPD management to facilitate earlier identification of high-risk patients and to guide multidisciplinary interventions. Prospective studies are warranted to determine whether targeted nutritional optimization can translate into meaningful reductions in exacerbation frequency and improved long-term outcomes. Further studies are needed to determine whether additional stratification within GOLD Group E has clinical implications for individualized risk assessment and management.

## Figures and Tables

**Table 1:** Demographic, Clinical, and Inflammatory Characteristics of the Study Population.

	All Subjects (n = 302) Mean ± SD	Age < 65 (n = 103) Mean ± SD	Age ≥ 65 (n = 199) Mean ± SD	*p*-Values
**Demographics**				
Age, year	67.85 ± 9.58	57.36 ± 6.15	73.29 ± 5.74	**<0.001**
Gender, Female, n (%)	80 (26.50%)	29 (28.20%)	51 (25.60%)	0.640
Body Weight, kg	74.81 ± 15.45	76.22 ± 14.39	74.08 ± 15.96	0.194
Height, cm	167.88 ± 8.13	169.53 ± 7.45	167.03 ± 8.36	0.005
BMI, kg/m^2^	26.53 ± 5.12	26.56 ± 4.87	26.52 ± 5.26	0.951
**Clinical characteristics**				
CAT Score	23.22 ± 8.79	19.31 ± 8.80	25.24 ± 8.09	**<0.001**
CCI	4.31 ± 3.11	3.02 ± 2.50	4.97 ± 3.20	**<0.001**
mMRC	2.47 ± 1.08	1.96 ± 0.96	2.73 ± 1.04	**<0.001**
Diagnosis duration, years	9.26 ± 6.78	5.45 ± 3.53	11.23 ± 7.21	**<0.001**
Smoking Status				**<0.001**
Smoker	150 (49.7%)	70 (68.0%)	80 (40.2%)	
Former Smoker	137 (45.4%)	28 (27.2%)	109 (54.8%)	
Non-Smoker	15 (5.0%)	5 (4.9%)	10 (5.0%)	
Pack-years	46.55 ± 20.74	40.15 ± 20.46	51.30 ± 19.74	**<0.001**
Exacerbations per 1 year	3.11 ± 2.33	2.43 ± 1.77	3.46 ± 2.50	**<0.001**
**Pulmonary Function Tests**				
FEV_1_% pred	50.45 ± 18.16	54.55 ± 18.40	48.27 ± 17.69	**0.004**
FEV_1_ (L)	1.46 ± 0.64	1.73 ± 0.70	1.31 ± 0.56	**<0.001**
FVC% pred	67.11 ± 19.83	70.88 ± 19.38	65.11 ± 19.82	**0.017**
FVC (L)	2.47 ± 0.92	2.82 ± 0.95	2.29 ± 0.85	**<0.001**
FEV_1_/FVC	58.30 ± 10.18	60.4 ± 9.83	57.18 ± 10.21	**0.003**
**Stable Period Biomarkers**				
Neutrophil count (×10^3^/μL)	6.30 ± 3.31	6.15 ± 4.08	6.38 ± 2.84	0.066
Lymphocyte count (×10^3^/μL)	1.86 ± 1.34	1.97 ± 1.14	1.81 ± 1.44	0.144
Platelet count (×10^3^/μL)	264.73 ± 88.80	271.09 ± 83.60	261.43 ± 91.40	0.223
Serum CRP (mg/L)	6.27 ± 13.10	4.76 ± 8.73	7.06 ± 14.83	**0.019**
Serum albumin (g/L)	39.17 ± 5.40	40.47 ± 5.10	38.49 ± 5.44	**0.002**
Serum protein (g/L)	65.89 ± 7.71	66.93 ± 7.78	65.35 ± 7.63	0.118
**Exacerbation Period Biomarkers**				
Neutrophil count (×10^3^/μL)	9.41 ± 5.18	9.40 ± 6.77	9.42 ± 4.13	0.274
Lymphocyte count (×10^3^/μL)	1.87 ± 1.85	2.09 ± 1.79	1.76 ± 1.87	**0.016**
Platelet count (×10^3^/μL)	271.27 ± 85.36	282.46 ± 78.79	265.48 ± 88.2	**0.048**
Serum CRP (g/dL)	50.95 ± 60.93	37.81 ± 47.67	57.75 ± 65.86	**0.023**
Serum albumin (g/L)	38.30 ± 5.44	39.76 ± 5.48	37.55 ± 5.28	**0.001**
Serum protein (g/L)	65.79 ± 7.86	66.81 ± 7.79	65.25 ± 7.86	0.095
**Medication Use**				
SABA/SAMA	237 (79%)	85 (82.50%)	152 (76.40%)	0.220
LAMA	58 (19%)	26 (25.20%)	32 (16.10%)	0.060
LABA/LAMA	86 (29%)	30 (29.10%)	56 (28.10%)	0.860
LABA/ICS	76 (25%)	25 (24.30%)	51 (25.60%)	0.800
LABA/LAMA/ICS	114 (38%)	32 (31.10%)	82 (41.20%)	0.080
LTOT	94 (31%)	20 (19.40%)	74 (37.20%)	**<0.001**
Nebulizer	173 (57%)	34 (33.00%)	139 (69.80%)	**<0.001**
**Inflammatory and Nutritional Indices**				
Baseline SII	1246.69 ± 1404.92	1135.42 ± 1152.68	1304.29 ± 1518.66	0.205
Exacerbation SII	2113.29 ± 2211.63	2080.78 ± 2618.45	2130.12 ± 1975.58	0.080
Baseline PNI	39.17 ± 5.40	40.48 ± 5.10	38.50 ± 5.44	**0.002**
Exacerbation PNI	38.31 ± 5.44	39.77 ± 5.48	37.56 ± 5.28	**<0.001**
Baseline NLR	4.55 ± 4.12	4.11 ± 3.82	4.78 ± 4.26	**0.040**
Exacerbation NLR	7.89 ± 7.52	7.54 ± 9.27	8.07 ± 6.45	**0.005**
Baseline GNRI	108.36 ± 13.35	110.48 ± 11.56	107.26 ± 14.09	**0.017**
Exacerbation GNRI	107.07 ± 13.33	109.42 ± 12.15	105.86 ± 13.77	**0.028**

FEV_1_, Forced expiratory volume in 1 s; FVC, Forced vital capacity; CCI, Charlson Comorbidity Index; CAT, COPD Assessment Test; mMRC, Modified Medical Research Council dyspnea scale; CRP, C-reactive protein; SII, Systemic immune-inflammation index; PNI, Prognostic nutritional index; NLR, Neutrophil-to-lymphocyte ratio; GNRI, Geriatric nutritional risk index; SD, standard deviation; LAMA, Long-acting muscarinic antagonist; LABA, Long-acting beta-agonist; ICS, Inhaled corticosteroid; LTOT, Long-term oxygen therapy; SABA, Short-acting beta-agonist; SAMA, Short-acting muscarinic antagonist. Comparisons between groups were performed using independent *t*-tests, Mann–Whitney U tests, or chi-square tests, as appropriate.

**Table 2:** Comparison of Clinical Indexes Between Baseline and Exacerbation Periods According to Age Groups.

Indexes	<65 (n = 103) Mean ± SD			≥65 (n = 199) Mean ± SD		
	Baseline	Exacerbation	*p*-Values	Baseline	Exacerbation	*p*-Values
**SII**	1135.42 ± 1152.68	2080.78 ± 2618.45	**<0.001**	1304.29 ± 1518.66	2130.12 ± 1975.58	**<0.001**
**PNI**	40.48 ± 5.10	39.77 ± 5.48	**0.035**	38.50 ± 5.44	37.56 ± 5.28	**<0.001**
**NLR**	4.11 ± 3.82	7.54 ± 9.27	**<0.001**	4.78 ± 4.26	8.07 ± 6.45	**<0.001**
**GNRI**	110.48 ± 11.56	109.42 ± 12.15	**0.029**	107.26 ± 14.09	105.86 ± 13.77	**<0.001**

SII, Systemic immune-inflammation index; PNI, Prognostic nutritional index; NLR, Neutrophil-to-lymphocyte ratio; GNRI, Geriatric nutritional risk index; SD, Standard deviation.

**Table 3:** Inflammatory and Nutritional Markers in Patients with ≤2 vs. >2 Exacerbations.

Indexes	≤2 Exacerbations (n = 150) Mean ± SD	>2 Exacerbations (n = 152) Mean ± SD	*p*-Values
Baseline SII	1017.47 ± 812.52	1472.90 ± 1783.07	0.108
Exacerbation SII	1824.34 ± 1988.30	2398.44 ± 2384.21	**0.010**
Baseline PNI	40.33 ± 5.27	38.04 ± 5.31	**<0.001**
Exacerbation PNI	39.07 ± 5.60	37.56 ± 5.20	**0.011**
Baseline NLR	3.84 ± 3.21	5.26 ± 4.76	**0.007**
Exacerbation NLR	6.59 ± 6.85	9.18 ± 7.94	**<0.001**
Baseline GNRI	109.09 ± 11.57	107.63 ± 14.91	0.342
Exacerbation GNRI	107.23 ± 11.99	106.92 ± 14.56	0.841

SII, Systemic immune-inflammation index; PNI, Prognostic nutritional index; NLR, Neutrophil-to-lymphocyte ratio; GNRI, Geriatric nutritional risk index; SD, Standard deviation.

**Table 4:** Negative Binomial Regression Analysis for Predictors of Annual COPD Exacerbations.

Variables	Exp(B)	95% Exp(B) CI Lower	95% Exp(B) CI Upper	*p*-Values
Age	0.999	0.992	1.007	0.880
Gender	0.987	0.850	1.150	0.865
FEV_1_%	0.987	0.982	0.991	**<0.001**
CAT Score	1.032	1.022	1.041	**<0.001**
Baseline PNI	0.986	0.973	0.999	**0.034**
Baseline NLR	1.028	1.012	1.042	**<0.001**

Statistically significant values (*p* < 0.05) are highlighted. Exp(B), exponentiated coefficient; CI, confidence interval; CAT, Chronic Obstructive Pulmonary Disease Assessment Test; PNI, Prognostic Nutritional Index; NLR, Neutrophil-to-lymphocyte ratio; FEV_1_, forced expiratory volume in 1 s.

## Data Availability

The data supporting the findings of this study are not publicly available due to ethical and institutional restrictions regarding patient confidentiality. However, anonymized data may be made available from the corresponding author upon reasonable request.
